# Gene expression analysis of vascular pathophysiology related to anti-TNF treatment in rheumatoid arthritis

**DOI:** 10.1186/s13075-019-1862-6

**Published:** 2019-04-15

**Authors:** Szilárd Póliska, Timea Besenyei, Edit Végh, Attila Hamar, Anita Pusztai, Andrea Váncsa, Nóra Bodnár, Szilvia Szamosi, Mária Csumita, György Kerekes, Zoltán Szabó, Zoltán Nagy, Gabriella Szűcs, Sándor Szántó, Gábor Zahuczky, László Nagy, Zoltán Szekanecz

**Affiliations:** 1 Department of Biochemistry and Molecular Biology, University of Debrecen Faculty of Medicine, Debrecen, Hungary; 2 Department of Rheumatology, University of Debrecen Faculty of Medicine, Nagyerdei str 98, Debrecen, 4032 Hungary; 3 Department of Internal Medicine, University of Debrecen Faculty of Medicine, Debrecen, Hungary; 4 Department of Angiology, University of Debrecen Faculty of Medicine, Debrecen, Hungary; 5 Department of Sports Medicine, University of Debrecen Faculty of Medicine, Debrecen, Hungary; 6 UD Genomed Ltd., Debrecen, Hungary

**Keywords:** Rheumatoid arthritis, Etanercept, Certolizumab pegol, Gene expression, Genetic signature, Atherosclerosis, Vascular pathology, Prediction, Response

## Abstract

**Objectives:**

Impaired vascular pathophysiology and increased cardiovascular (CV) mortality are associated with rheumatoid arthritis (RA). To date, no genomic analysis of RA- and RA treatment-related vascular pathophysiology has been published. In this pilot study, we performed gene expression profiling in association with vascular pathophysiology in RA patients.

**Methods:**

Sixteen and 19 biologic-naïve RA patients were included in study 1 and study 2, respectively. In study 1, genetic signatures determined by microarray were related to flow-mediated vasodilation (FMD), pulse-wave velocity (PWV), and common carotid intima-media thickness (IMT) of patients. In study 2, clinical response (cR) vs non-response (cNR) to 1-year etanercept (ETN) or certolizumab pegol (CZP) treatment, as well as “vascular” response (vR) vs non-response (vNR) to biologics, were also associated with genomic profiles. Multiple testing could not be performed due to the relatively small number of patients; therefore, our pilot study may lack power.

**Results:**

In study 1, multiple genes were up- or downregulated in patients with abnormal vs normal FMD, IMT, and PWV. In study 2, there were 13 cR and 6 cNR anti-tumor necrosis factor (TNF)-treated patients. In addition, 10, 9, and 8 patients were FMD-20%, IMT-20%, and PWV-20% responders. Again, vascular responder status was associated with changes of the expression of various genes. The highest number of genes showing significant enrichment were involved in positive regulation of immune effector process, regulation of glucose transport, and Golgi vesicle budding.

**Conclusion:**

Differential expression of multiple genetic profiles may be associated with vascular pathophysiology associated with RA. Moreover, distinct genetic signatures may also be associated with clinical and vascular responses to 1-year anti-TNF treatment.

**Electronic supplementary material:**

The online version of this article (10.1186/s13075-019-1862-6) contains supplementary material, which is available to authorized users.

## Key messages

- Rheumatoid arthritis is associated with impaired vascular pathophysiology
- Endothelial dysfunction and atherosclerosis may be associated with altered genetic signature
- Anti-TNF therapy may improve vascular function associated by changes in gene expression

## Introduction

Accelerated atherosclerosis and increased cardiovascular (CV) morbidity and mortality have been associated with rheumatoid arthritis (RA). Vascular pathophysiology found in RA has been characterized by endothelial dysfunction, increasing arterial stiffness and overt atherosclerosis. These alterations are indicated by impaired brachial artery flow-mediated vasodilation (FMD), arterial pulse-wave velocity (PWV), and carotid plaque plus carotid intima-media thickness (IMT), respectively [1–4]. Several other non-invasive techniques have been available to detect vascular pathophysiology [1]. All these changes can be preclinically detected in RA with a negative history of CV disease (CVD) [2–6].

Targeted therapies including tumor necrosis factor α (TNF-α) inhibitors are highly effective in RA [7, 8]. The efficacy of these biologics may differ from patient to patient. Therefore, there is a high need for the identification of biomarkers including genetic signatures that may predict therapeutic response (R) vs non-response (NR) to biologics [9–12]. Indeed, gene expression profiling has been successfully used on tissue samples or blood for the identification of biomarkers and/or genome classifiers in various diseases [13, 14]. As described by several investigators, gene expression patterns of peripheral blood mononuclear cells (PBMCs) may be associated with response to therapies or disease progression [9–12, 15]. We have also performed gene expression studies and identified certain genomic signatures that may be associated with responses to infliximab [16] and tocilizumab [17]. Thus, we developed a standard protocol that could utilize genomics as biomarkers of disease or therapeutic outcomes [16, 17].

Both RA and atherosclerosis have strong genetic backgrounds. In RA, genome-wide association studies (GWAS) revealed numerous susceptibility alleles including *HLADRB1*, *PTPN22*, *TRAF1/C5*, *STAT4*, *PADI4*, *IRF5*, *FCGR*, *IL2RA*, *IL2RB*, *CD40*, *CCR6*, and *CCL21.* More than 40 single nucleotide polymorphisms (SNPs) have been associated with RA [18]. In atherosclerosis, a GWAS study carried out on more than 100,000 Europeans revealed association of more than 30 genes with coronary atherosclerosis. These included matrix molecule (e.g., *ADAMTS7*, *ANKS1A*, *COL4A1*), lipid (e.g., *LPA*, *LDLR*), chemokine (e.g., *CXCL12*), and other genetic loci [19]. The roles of other “lipid-related genes” including *ABCA1*, *APOA5*, *LCAT*, *CETP*, and *SORT1*, as well as more than 50 “non-lipid” genes, have also been identified in atherosclerosis by GWAS [20]. *HLADRB1* has been associated with both RA [18] and CVD [19].

It is also possible that RA and the associated CVD may have common genetic denominators. In this respect, mostly single-allele studies have been performed. Farragher et al. [21] reported that certain HLA-DRB1 alleles, mainly those functioning as shared epitope (SE), are associated with increased CV mortality in RA. For example, in comparison to RA patients carrying no or one SE allele, those with HLA-DRB1*01/*04 exert a three times higher risk for CV death [21]. Gonzalez-Gay et al. [22] also confirmed a relationship between SE alleles and CV morbidity and mortality. Studies on single non-HLA alleles were primarily performed by Gonzalez-Gay et al. [23, 24]. For example, the A1298C SNP in the *MTHFR* gene was associated with occurrence of CV events in RA patients. A SNP in the *SMAD3* gene increased the risk for cerebrovascular accident in ACPA-negative RA patients. The HLA-DR1*04/*04 genotype and an SNP in the *CD40* gene were associated with endothelial dysfunction and IMT, respectively. SNPs in the *IRF5* and osteoprotegerin (*OPG*) genes could also be associated with vascular pathology. On the other hand, no associations between atherosclerosis/CVD or SNPs in the *IFNG*, *JAK3*, *PON1*, *ADAMTS7*, *CARD8*, *CXCL12*, *ADIPOQ*, and *TLR4* alleles could be demonstrated [23, 24]. Yet, association of RA and atherosclerosis with complex genetic signatures has not yet been elucidated.

Therefore, we wished to determine associations between clinical responses to biologics, vascular pathophysiology, and gene expression patterns in a pilot study. Here, we performed global gene expression profiling in PBMCs of RA patients. We associated gene expression profiles (signatures) with (1) normal vs abnormal FMD, PWV, and IMT status of patients (study 1), (2) clinical response (cR) vs non-response (cNR) to 1-year biologic (etanercept, ETN, or certolizumab pegol, CZP) treatment as defined by EULAR response criteria (study 2), and (3) “vascular” response (vR) vs non-response (vNR) as defined by sufficient changes in FMD, PWV, and/or IMT upon 1-year ETN or CZP therapy (study 2).

## Materials and methods

### Patients

In study 1, 16 Caucasian, biologic-naive RA patients (15 females, 1 male), with a mean age of 53.7 ± 5.7 (range 42–60) years and a mean disease duration of 10.0 ± 10.2 (range 2–44), were included. In study 2, 19 biologic-naïve RA patients (18 females, 1 male; mean age 54.3 ± 4.8 [range 43–60] years, 12.1 ± 10.9 [range 2–44] years) were recruited. Later, 12 patients received ETN, and 7 CZP. All RA patients met the 2010 EULAR/ACR classification criteria for RA [25].

All blood samples were obtained after the subjects fasted overnight for 12 h locally between 8:00 AM and 9:00 AM before the first admission of biologics. Medication remained unchanged during the study.

The inclusion criteria in both studies included confirmed diagnosis of RA, age between 20 and 60, failure to respond to at least two DMARDs, active disease (DAS28 > 3.2), and anti-TNF therapy-naïve patients. Corticosteroid therapy (prednisone ≤ 10 mg per day) was allowed provided that the dosage had been stable for at least 2 months before entry. Also non-steroidal anti-inflammatory drugs (NSAID) were allowed in doses stable for at least 1 month before baseline. All patients received 10–25 mg/week oral methotrexate (MTX) treatment, which had been stable for at least 4 weeks before baseline. Exclusion criteria included pregnancy or breastfeeding, current or recent malignancies, active infectious disease, patients with a history of arthritis or connective tissue disease other than RA, and smoking.

Disease activity was assessed by determining the 28-joint Disease Activity Score (DAS28) at baseline and then 12 months after the initiation of anti-TNF treatment in study 2.

Clinical responder (cR vs cNR) status was determined after 12 months of treatment with either ETN or CZP by the EULAR response criteria originally described by Van Gestel et al. [26].

The Medical Research Council of Hungary gave ethical approval for this study (No. 9732-2/2012/EHR). In addition, the Institutional Review Board of the University of Debrecen Faculty of Medicine also approved the protocol. The study was in compliance with the Helsinki Declaration. Signed informed consent was obtained from all individuals providing blood samples.

### Assessment of vascular physiology by ultrasound

Brachial artery FMD was assessed as described before [2, 27, 28]. In brief, ultrasound examination was performed on the right arm using a 10-MHz linear array transducer (ultrasound system: HP Sonos 5500) by a single trained sonographer after 30  min of resting in a temperature-controlled room (basal value for FMD). A B-mode longitudinal section was obtained of the brachial artery above the antecubital fossa. In order to assess FMD, reactive hyperemia was induced by release of a pneumatic cuff around the forearm inflated to suprasystolic pressure for 4.5 min. After deflation, the maximal flow velocity and the arterial diameter was continuously recorded for 90 s. Flow velocities, the baseline diameter, and FMD were ECG gated and detected offline. FMD values were expressed as % change from baseline (resting) value (FMD%). In our previous work, we divided RA patients into “high (normal) FMD” and “low (impaired) FMD” subsets by defining a cutoff value of 5% [2]. We used the same cutoff in the present studies.

The IMT measurements were carried out as described before [2, 28, 29]. Briefly, a duplex ultrasound system (HP Sonos 5500, 10 MHz linear array transducer) was used to assess the common carotid arteries by a single observer. Longitudinal high-resolution B-mode ultrasound scans were employed over both the right and left common carotid arteries and were R-synchronized and recorded. The offline measurements were performed 1 cm proximal to the carotid bulb in the far wall. IMT was defined as the distance between the first and second echogenic lines from the lumen taking the average of 10 measurements on both sides. IMT values were expressed in millimeters. In our previous work, we divided RA patients into “high (increased) IMT” and “low (normal) IMT” subsets by using a cutoff value of 0.65 mm [2]. We used the same cutoff in the present studies.

With respect to arterial stiffness, PWV was calculated automatically by a TensioClinic arteriograph system (Tensiomed Ltd., Budapest, Hungary) with the quotient of the distance between the jugular fossa and symphysis as described before [28, 30, 31]. If an artery is elastic, PWV is low. With decreased arterial elasticity, PWV rises. The arteriograph assesses this parameter from the oscillometric data obtained from the 35 mmHg suprasystolic pressure of the brachial artery [30, 31]. In order to obtain reproducible results, the patient had to rest in a supine position for at least 10  min before the assessment in a quiet room. PWV is expressed in meters per second. Based on our previous experience [28, 31], we used a cutoff value of 8 m/s, where PWV ≤ 8 m/s and PWV > 8 m/s indicated “low (normal)” and “high (increased)” PWV, respectively.

In study 2, we defined the arbitrary vascular responder status (vR vs vNR). Patients achieving at least 20% improvement in FMD, IMT, or PWV were considered vascular responders (vR). Patients achieving ≥ 20% improvement in at least two out of the three vascular parameters (FMD, IMT, PWV) were considered good vascular responders (GVR).

### PBMC and RNA isolation

Venous peripheral blood samples were collected (10 ml) in Venous Blood Vacuum Collection Tubes containing EDTA (BD Vacutainer K2EDTA). PBMCs were separated by Ficoll gradient centrifugation. Total RNA was extracted from PBMCs using Trizol reagent (Invitrogen), according to the manufacturer’s protocol. RNA quality was checked on an Agilent Bioanalyzer 2100 (Agilent Technologies); all samples had a 28S/18S ratio between 1.5 and 2.0, and the RNA Integrity Number was between 9 and 10. Quantity was determined by NanoDrop (Thermo Scientific).

### Microarray analysis and statistics

Affymetrix GeneChip Human Primeview array was used to analyze global expression pattern of 28,869 well-annotated genes. 3’ IVT Expression Kit (Affymetrix) and GeneChip WT Terminal Labeling and Control Kit (Affymetrix) were used for amplifying and labeling 250 ng of RNA samples. Samples were hybridized at 45 °C for 16 h, and then standard washing protocol was performed using a GeneChip Fluidics Station 450 and the arrays were scanned on a GeneChip Scanner 7G (Affymetrix).

Microarray data were analyzed by Genespring GX12 software (Agilent Technologies). Affymetrix data files were imported using the RMA algorithm and median normalization was performed. To identify differentially expressed genes between clinical conditions, statistical analysis was performed using a Mann-Whitney *U* test; *p* value < 0.05 was considered to significant difference. We used hierarchical clustering and principal component analysis (PCA) to show the separation of clinical conditions by the differentially expressed genes. Microarray data were submitted to Gene Expression Omnibus (GEO), accession number: GSE126476.

Gene Ontology (GO) analysis was performed using Cytoscape 3.4.0 software (cytoscape.org) with the ClueGO application. The settings were the following: GO biological process, GO immune system process, and KEGG human diseases pathways; statistical options: two-sided hypergeometric test and Benjamini-Hochberg FDR for multiple testing correction. Significantly enriched GO categories were considered to *p* value < 0.05 and *κ* score < 0.4.

The association between clinical and various vascular responses was analyzed by Pearson’s correlation (*p* < 0.05).

## Results

### Gene expression profiles may differentiate patients with normal vs impaired vascular pathophysiology

**Fig. 1:**
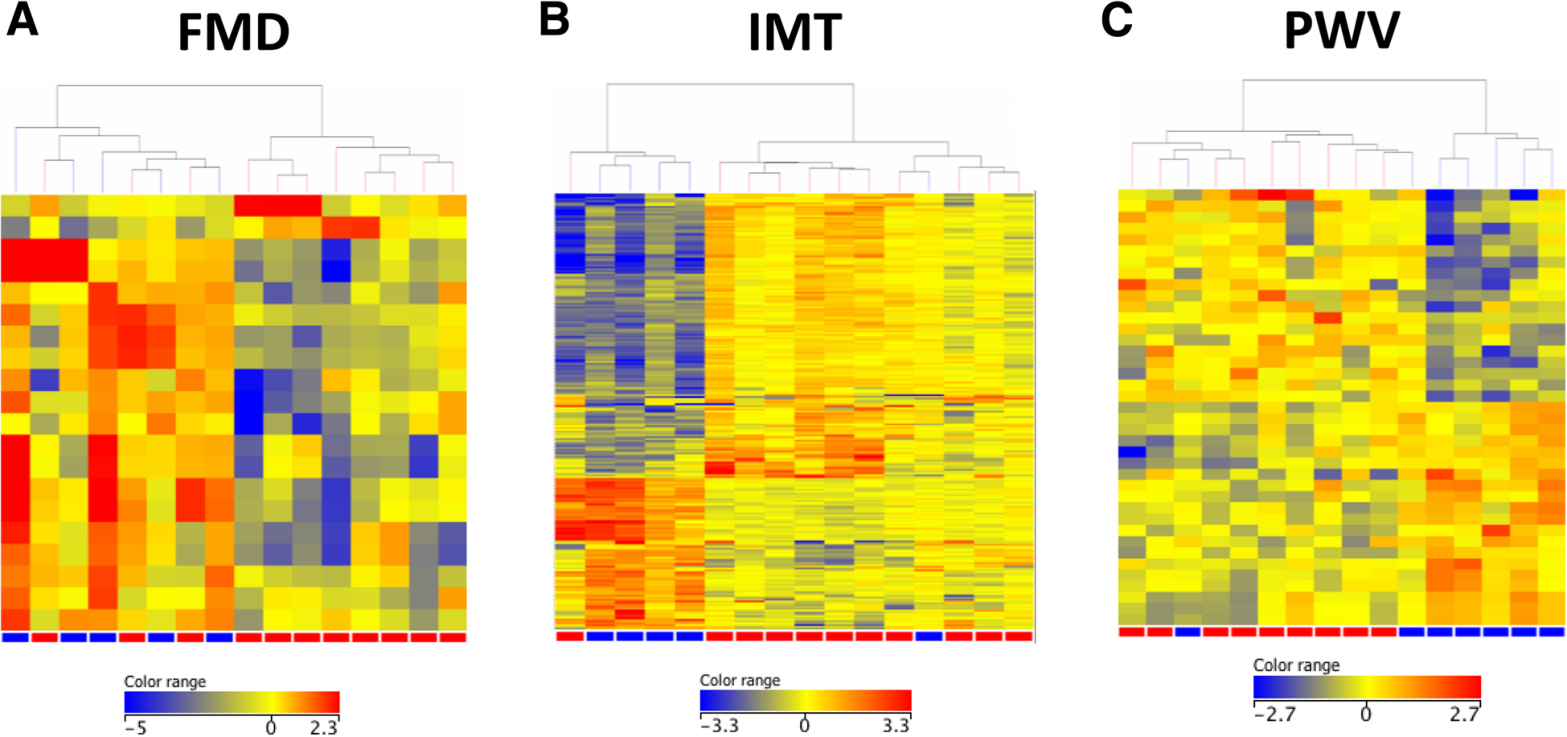
Study 1. Differential gene expression associated with vascular pathophysiology in RA. The heatmaps indicate differential gene regulation in patients with low (red) versus high (blue) FMD (**a**), IMT (**b**), and PWV (**c**). Among the 16 patients, 11 had impaired (low; red) and 5 normal (high; blue) FMD (**a**). Eleven patients had normal (low; red) and 5 had increased (high; blue) IMT (**b**). Nine patients had normal (low; red) and 7 had increased (high; blue) PWV (**c**). See Table S1 for the actual gene data

In study 1, among the 16 RA patients, 11 had low (< 5%; red in Fig. 1a, d) and 5 had high (≥ 5%; blue) FMD values (Fig. 1a). Similarly, low (≤ 0.65 mm; red in Fig. 1b, e) and high (> 0.65 mm; blue) IMT values were observed in 11 and 5 patients, respectively (Fig. 1b, e). PWV was low (≤ 8 m/s; red in Fig. 1c, f) in 9 and high (> 8 m/s; blue) in 7 patients (Fig. 1c, f).

Two genes (*CD74*, *ZNF718*) were up- and 12 genes were significantly downregulated in patients with impaired (low) FMD compared to those with normal (high) FMD. The 12 downregulated genes are *FOLR3*, *ADM*, *HP*, *DSC2*, *ANXA3*, *LILRA5*, *PLSCR1*, *AKAP12*, *VNN2*, *TCN1*, *HDC*, and *NFIL3* (Additional file 1: Table S1; Fig. 1d).

Altogether 62 up- and 129 downregulated genes were associated with increased (high) versus normal (low) IMT. The upregulated genes included *G0S2*, *NRGN*, *ITGA2B*, *C3*, *FLNA*, *IRF5*, *ABCC3*, *CAPNS1*, *IL2RG*, *CCL4L1*, *ACTN1*, *HLAB*, *HLAC*, *TNFAIP3*, and *MYO1G.* Among others, *PPP1CB*, *HLADRB4*, *IFNGR1*, *LRRN3*, *CCR2*, *CD46*, *IFI44L*, *IFIT1*, *TLR10*, *CD164*, *IFIT2*, *SMAD4*, and *SGPP1* genes were downregulated (Additional file 1: Table S1; Fig. 1e).

Finally, 32 genes showed differential expression between patients with increased (high) compared to normal (low) PWV. The changes were slight in most of these genes, and only two genes showed ≥ 2-fold change difference between the high vs. low comparison, *HLAB/HLAC* (upregulated) and *LRRN3* (downregulated) (Additional file 1: Table S1; Fig. 1f).

### Association between clinical and vascular response upon 1-year anti-TNF therapy

**Fig. 2:**
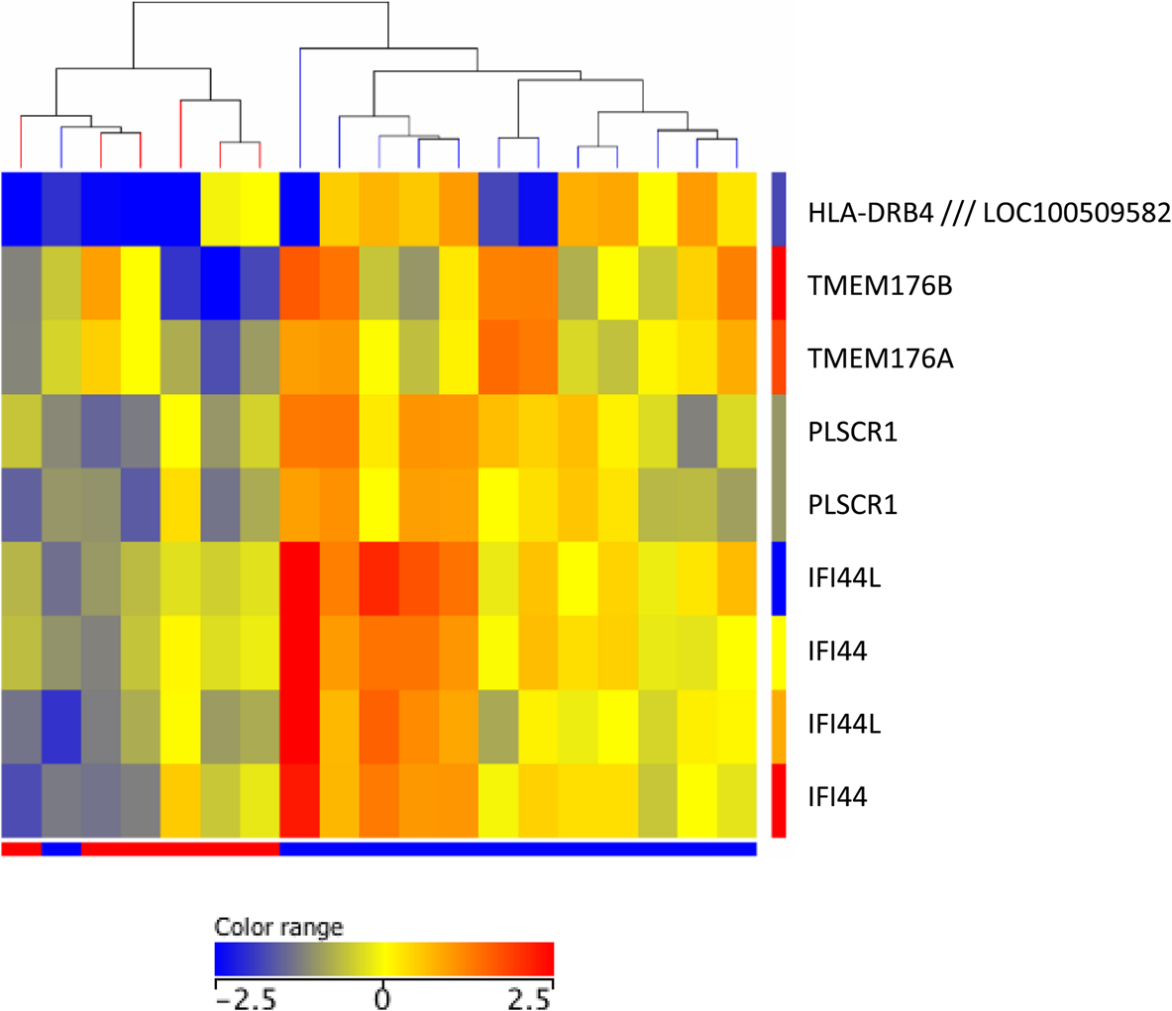
Study 2. Differential gene expression associated with clinical response or non-response after 12 months of anti-TNF therapy. Altogether, 13 patients were responders and 6 were non-responders. Heatmap indicates differential gene regulation in clinical responders (blue) vs non-responders (red)

**Fig. 3:**
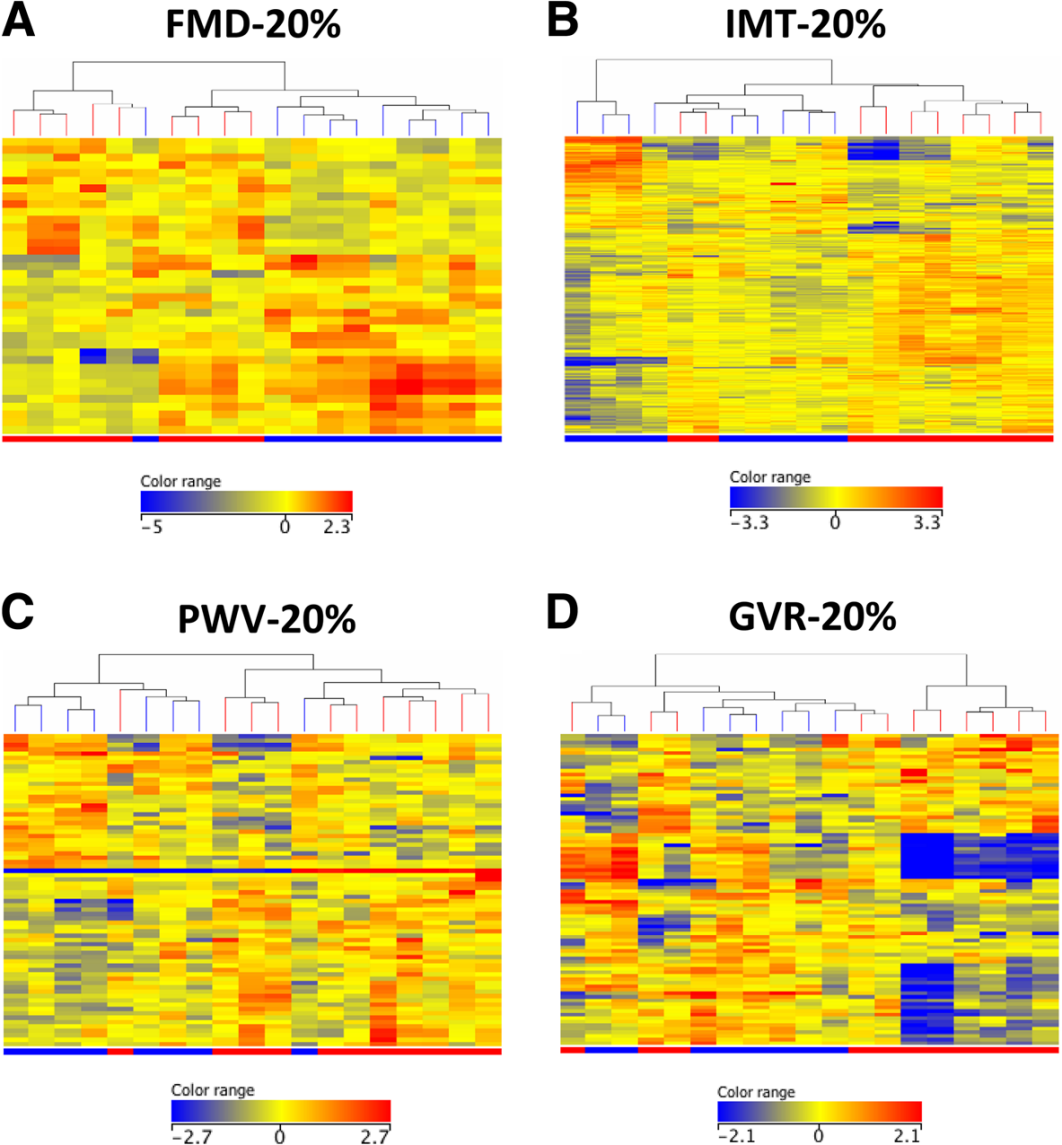
Study 2. Differential gene expression associated with vascular responses to 12-month anti-TNF therapy. Among the 19 patients, 10 showed FMD-20%, 9 demonstrated IMT-20%, 8 showed PWV-20%, and 8 exerted GVR-20% responses. Heatmaps indicate differential gene regulation in FMD-20% (**a**), IMT-20% (**b**), PWV-20% (**c**), and GVR-20% (**d**) responders (blue) vs non-responders (red). See Table S2 for the actual gene data

In study 2, 19 biologic-naïve RA patients were treated with either ETN or CZP for 12 months. Clinical (cR vs cNR) and vascular (vR vs vNR) responder status was assessed after 1 year as described above. There were 13 cR and 6 cNR patients (Table 1; Fig. 2; cR: blue, cNR: red). According to the arbitrary definition of vascular responder status described above, 10 patients were FMD-20% responders (Table 1; Fig. 3a), 9 were IMT-20% responders (Table 1; Fig. 3b), and 8 were PWV-20% responders (Table 1; Fig. 3c). Altogether, 8 patients achieved the GVR-20% status (Table 1; Fig. 3d) (in the figures, responders are in blue and non-responders are in red). Out of the 19 patients, 5 patients achieved response in all three vascular parameters (FMD, IMT, PWV), and 3 in two parameters.

**Table 1:** Clinical and vascular responder status in ETN- or CZP-treated RA patients (*n* = 19) (study 2)

Subject	Biologic	Clinical response	FMD response	IMT response	PWV response	Good vascular response
1	ETN	R	R	R	R	R
2	ETN	R	NR	R	NR	NR
3	CZP	R	R	R	NR	R
4	ETN	R	NR	NR	NR	NR
5	ETN	R	R	NR	NR	NR
6	ETN	R	R	NR	R	R
7	CZP	R	NR	R	NR	NR
8	ETN	R	R	R	R	R
9	CZP	R	R	R	R	R
10	ETN	R	NR	R	R	R
11	ETN	R	NR	NR	NR	NR
12	ETN	R	R	R	R	R
13	ETN	R	R	NR	NR	NR
14	CZP	NR	R	NR	NR	NR
15	ETN	NR	NR	NR	NR	NR
16	CZP	NR	NR	NR	NR	NR
17	CZP	NR	NR	NR	R	NR
18	CZP	NR	NR	NR	NR	NR
19	ETN	NR	R	R	R	R
R (*n*)		13	10	9	8	8
NR (*n*)		6	9	10	11	11

*R* responder, *NR* non-responder, *ETN* etanercept, *CZP* certolizumab pegol

Clinical and vascular responder status of the treated patients was also compared (Table 2). When clinical responder status was correlated with FMD, IMT, or PWV responder status, as well as the GVR status, IMT response showed a tendency of correlation with the clinical response (*R* = 0.418, *p* = 0.075) (Table 2). Yet, 8 FMD, 8 IMT, 6 PWV vRs, and 7 patients achieving GVR status were also cRs. On the other hand, 4, 5, 4, and 5 patients were both FMD, IMT, PWV, and global vascular NRs and cNRs, respectively (Table 2).

**Table 2:**
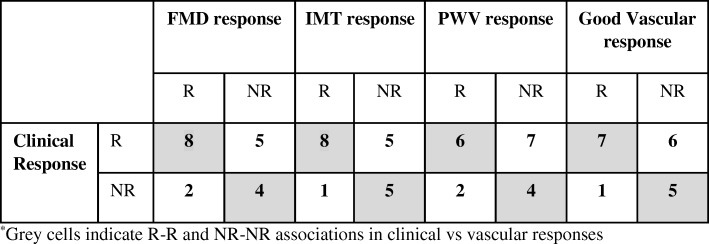
Association of clinical and vascular responses in ETN- or CZP-treated RA patients (n=19) (Study 2)^*^

### Differential baseline gene expression between clinical and vascular responders vs non-responders

First, baseline gene expression was associated with cR vs cNR status after 12 months of anti-TNF therapy (Fig. 2; Additional file 2: Table S2). Only 5 genes (*HLADRB4*, *TMEM176A*, *TMEM176B*, *IFI44*, and *PLSCR1*) were significantly (≥ 2-fold) upregulated in cR vs cNR patients (*p* < 0.05). No significantly downregulated genes were found (Fig. 2; Additional file 2: Table S2).

Regarding vascular responses (vR) to biologics, the associations of baseline genetic signatures with vR vs vNR after 1-year ETN or CZP therapy were determined with respect to FMD, IMT, PWV, and GVR. FMD-20% vR (≥ 20% improvement/increase in FMD after 12 months) vs vNR was associated with significant (≥ 2-fold) upregulation of a single gene (*NEFL*) and downregulation of two genes (*JUN* and *GYPB*) (*p* < 0.05) (Fig. 3a; Additional file 2: Table S2). IMT-20% vR (≥ 20% improvement/decrease in IMT after 12 months) vs vNR was associated with upregulation of 18 (e.g., various immunoglobulin and HLA genes, *TNFRSF17*, *CD74*, *FCRL5*, *CD79A*, *IFITM3*) and downregulation of 12 genes (e.g., *CXCL5*, *ITGB3*, *NEFL*) (Fig. 3b, Additional file 2: Table S2). PWV-20% vR (≥ 20% improvement/decrease in PWV after 12 months) vs vNR was associated with upregulation of three genes (*IFNG*, *JUN*, and *CCL4L1/L2*) and downregulation of five genes (*HLAC*, *GNB4*, *NRG1*, *NEFL*, and *FKBP5)* (Fig. 3c, Additional file 2: Table S2). Finally, a good vascular response (GVR-20%) was defined as improvement by ≥ 20% in at least two vascular parameters (increase in FMD, decrease in IMT or PWV). In this respect, GVR-20% vR vs vNR was associated with the upregulation of 11 genes (e.g., various immunoglobulin genes, *SCN3A*, *CD79A*, and *FCRL5*) and downregulation of two genes (*NEFL* and *CES1/CES1P1*) (Fig. 3d, Additional file 2: Table S2).

### Clustering and network analysis of differentially expressed genes

**Fig. 4:**
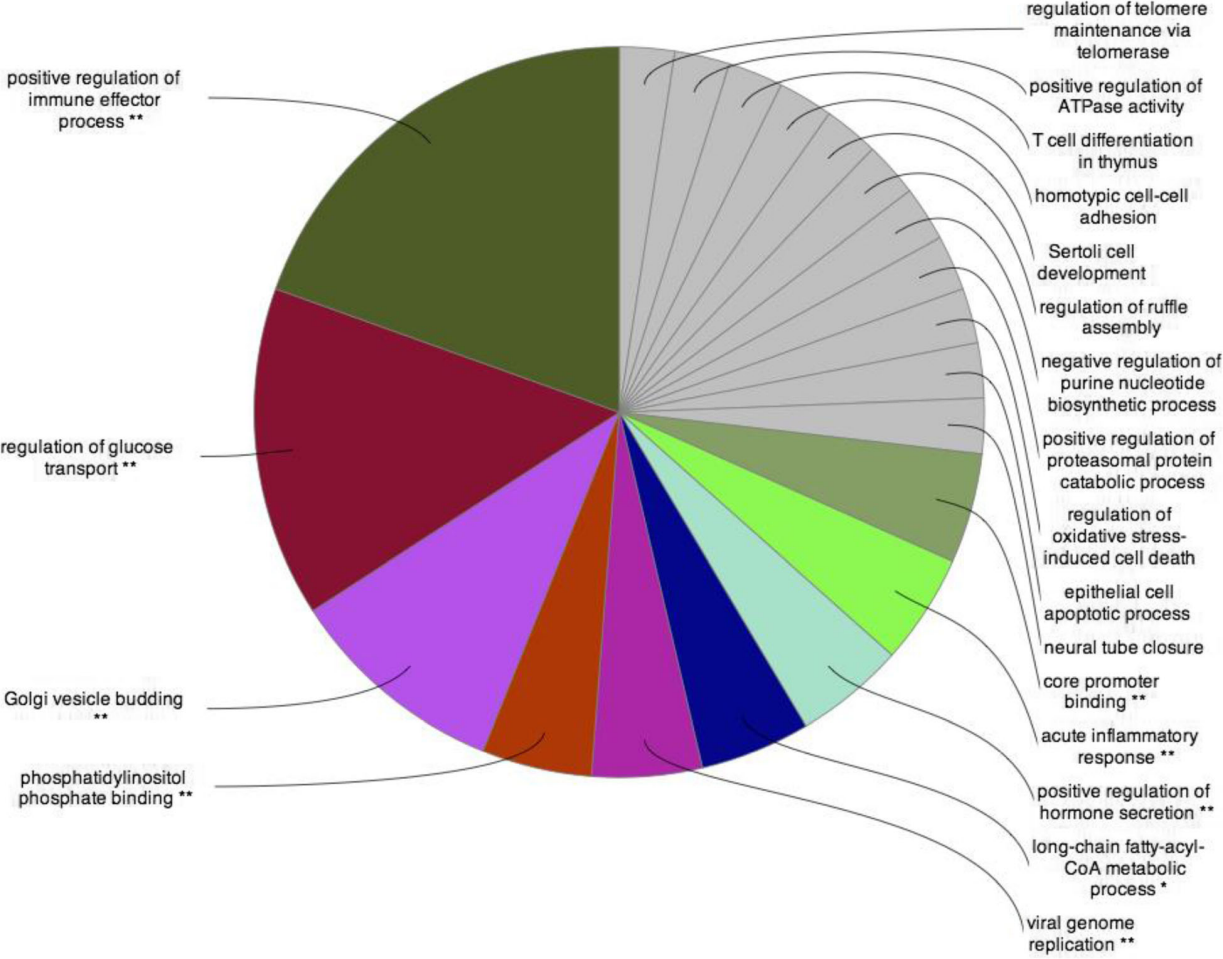
Pie chart shows the IMT-associated, overrepresented functional categories of the ClueGO pathway analysis (** *p* < 0.001,* *p* < 0.01, without star *p* < 0.05)

Gene Ontology (GO) analysis was performed in order to demonstrate functional categories of differentially expressed genes, which showed at least twofold change up- or downregulation in study 2. As IMT has been associated with differential expression of numerous genes, we performed GO on IMT-, but not on FMD- or PWV-associated genes. Figure 4 shows the overrepresented functional categories of differentially expressed IMT-associated genes. The highest number of genes showing significant enrichment was involved in positive regulation of immune effector process, regulation of glucose transport, Golgi vesicle budding, and others (Fig. 4).

## Discussion

Accelerated atherosclerosis, as well as increased CV morbidity and mortality, has been associated with RA [2, 4, 32, 33]. Several HLA and non-HLA genes have been implicated in susceptibility to RA [18, 34, 35] and atherosclerosis [19, 22, 36]. There may be common genetic factors underlying both RA and atherosclerosis [21, 23, 24]. Previous genetic studies mostly revealed the involvement of single SNPs in the background of RA-driven atherosclerosis. Thus, both HLA-DRB1 [21, 22, 37] and non-HLA alleles [23, 24] have been detected in this respect. To our knowledge, no complex genomic studies have been performed and published in association with CV pathophysiology or the effect of anti-TNF therapy on vascular function in RA. Therefore, we conducted a pilot study on a group of RA patients and compared clinical data, vascular pathophysiology, and patterns of differentially expressed genes.

Some genes were differentially regulated in association with FMD, IMT, and PWV or in conjunction to clinical and vascular responses to anti-TNF treatment. As discussed above, the Spanish group described numerous SNPs associated with RA-related CV disease [23, 24]. The genes identified by them, similarly to our study, encoded HLA, pro-inflammatory cytokines, and several other molecules related to autoimmunity and inflammation. As no complex genomic study or genetic study on biologic-treated patients have been conducted, our results cannot be compared to any previous studies.

In study 1, we applied pre-determined cutoff values for FMD, IMT, and PWV in order to define and to compare abnormal and normal vascular functions in 16 RA patients. A ≥ 2-fold change in gene expression was considered meaningful and significant. Some genes were differentially expressed in RA patients with abnormal (high) vs normal (low) IMT. Much fewer genes were associated with abnormal (low) FMD, and only one gene was up- and one gene was downregulated in patients with abnormal (high) PWV. Differentially expressed genes primarily included MHC-related genes, but also genes encoding cytokine, adhesion molecule, integrin, and interferon-related ones.

In study 2, we treated 19 RA patients with TNF inhibitors (either ETN or CZP) for 1 year. In the end, 13 patients showed a clinical response to treatment. We defined vascular responses as at least 20% improvement in FMD, IMT, and PWV after 12 months of biologic treatment. We also defined GVR as ≥ 20% improvement in at least two vascular parameters. Only 10–13 out of 19 patients had concordance between clinical and any vascular response. Clinical response was associated with the upregulation of five genes only. Again, some genes were differentially expressed in IMT responder RA patients compared to non-responders. Very few genes were associated with FMD, PWV, or GVR responses to anti-TNF therapy. Here, differentially expressed genes primarily included immunoglobulin- and HLA-related genes, but also other genes including cytokine, chemokine, and interferon-related genes.

A few particular genes were picked up in multiple analyses. Just to present a few interesting examples, two vanin genes (*VNN1* and *VNN2*) showed differential expression in study 1. Several interferon-related genes (e.g., *IFI44*, *IFIT1*, *IFITM3*, *IRF5*) exerted differential expression in both studies. In SNP studies, IRF5 genetic variants were associated with CV disease in RA [38]. Although the role of vanins in RA is unknown, these molecules have been implicated in fibrosis and vascular pathology underlying other autoimmune conditions [39] and atherosclerosis [40]. The gene for neurofilament light polypeptide (*NEFL*) was significantly associated with FMD, IMT, PWV, and GVR responses in study 2. Neurofilament proteins have been associated with nervous system damage in SLE [41]. However, cytoskeletal neurofilaments have also been detected in the RA synovium [42]. Antibodies to neurofilament proteins may play a role in RA, as well as SLE [43]. Leucine-rich repeat neuronal 3 (*LRRN3*) gene exerted differential expression in RA patients with abnormal IMT and PWV in study 1. This gene has been associated with aging [44]. In general, leucine-rich repeat kinases (LRRK) are involved in neuroinflammation, but also RA and other arthritides [45]. Finally, in study 2, *JUN*, the gene encoding the jun proto-oncogene, also showed differential expression in association with FMD-20% and PWV-20% responses. This molecule [46, 47], as well as c-Jun N-terminal kinase (JNK) [48, 49], has long been associated with the pathogenesis of RA and atherosclerosis. Certainly, several other genes and their roles in RA and/or CV disease could have been demonstrated.

## Conclusion

In order to demonstrate the function of the genes showing differential expression in association with RA-related vascular pathophysiology, GO analysis was performed on IMT-associated, overrepresented genes. In general, the differential expression of numerous genes was associated with FMD, IMT, and PWV in RA. Genetic signatures were also associated with clinical and vascular responses to anti-TNF therapy. The major strength of this study is that after SNP studies, this may be the first one studying more complex genetic signatures in relation to RA-associated atherosclerosis. Our study may have limitations including the relatively low number of patients and that multiple testing could not be carried out, so this study may lack power. Therefore, after this pilot study, further, larger ones need to be conducted in order to further delineate the genetic/genomic background of RA-related vascular disease.

## Additional files

Additional file 1: **Table S1.** Association of gene expression profiles with vascular pathophysiology in biologic-naïve RA patients (*n* = 16) (study 1). (DOCX 35 kb)

Additional file 2: **Table S2.** Association of gene expression profiles with clinical and vascular responses upon anti-TNF therapy in RA patients (*n* = 19) (study 2). (DOCX 22 kb)

## Abbreviations

ACPA: Anti-citrullinated peptide antibody
ACR: American College of Rheumatology
cNR: Clinical non-response/responder
cR: Clinical response/responder
CV: Cardiovascular
CVD: Cardiovascular disease
CZP: Certolizumab pegol
DAS28: 28-Joint disease activity scale
DMARD: Disease-modifying antirheumatic drug
ETN: Etanercept
EULAR: European League Against Rheumatism
FMD: Flow-mediated vasodilation
GO: Gene Ontology
GVR: Good vascular response/responder
GWAS: Genome-wide association study
HLA: Human leukocyte antigen
IMT: Intima-media thickness
MTX: Methotrexate
NR: Non-response
NSAID: Non-steroidal anti-inflammatory drug
PBMC: Peripheral blood mononuclear cell
PWV: Pulse-wave velocity
R: Responder
RA: Rheumatoid arthritis
SE: Shared epitope
SLE: Systemic lupus erythematosus
SNP: Single nucleotide polymorphism
TNF-α: Tumor necrosis factor alpha
vNR: Vascular non-response/responder
vR: Vascular response/responder
