# Supplementary material for: Gene expression analysis of vascular pathophysiology related to anti-TNF treatment in rheumatoid arthritis
Source: Arthritis Res Ther. 2019 Apr 15;21:94. doi: 10.1186/s13075-019-1862-6 (PMC6466794; doi:10.1186/s13075-019-1862-6)
Supplement: Supplementary file 1 — Table S1. Association of gene expression profiles with vascular pathophysiology in biologic-naïve RA patients (n = 16) (study 1). (DOCX 35 kb) [file 13075_2019_1862_MOESM1_ESM.docx]

**Table S1.** Association of gene expression profiles with vascular pathophysiology in biologic-naive RA patients (n=16) (Study 1)

| **Up-regulated genes (≥2-fold)** | | | | **Down-regulated genes (≥2-fold)** | | | |
| --- | --- | --- | --- | --- | --- | --- | --- |
| ***Gene symbol*** | ***Gene title*** | ***FC(abs)*** | ***p*** | ***Gene symbol*** | ***Gene title*** | ***FC(abs)*** | ***p*** |
|  | | | | | | | |
| *FMD (impaired/low vs normal/high)* | | | | | | | |
|  | | | | | | | |
| *CD74* | CD74 molecule, major histocompatibility complex, class II invariant chain | 2.093 | 0.027 | *FOLR3* | folate receptor 3 | 3.693 | 0.036 |
| *ZNF718* | zinc finger protein 718 | 2.029 | 0.047 | *ADM* | adrenomedullin | 3.214 | 0.027 |
|  |  |  |  | *HP* | haptoglobin | 2.661 | 0.047 |
|  |  |  |  | *DSC2* | desmocollin 2 | 2.621 | 0.020 |
|  |  |  |  | *ANXA3* | annexin A3 | 2.575 | 0.036 |
|  |  |  |  | *LILRA5* | leukocyte immunoglobulin-like receptor, subfamily A (with TM domain), member 5 | 2.368 | 0.020 |
|  |  |  |  | *PLSCR1* | phospholipid scramblase 1 | 2.198 | 0.015 |
|  |  |  |  | *AKAP12* | A kinase (PRKA) anchor protein 12 | 2.122 | 0.036 |
|  |  |  |  | *VNN2* | vanin 2 | 2.110 | 0.036 |
|  |  |  |  | *TCN1* | transcobalamin I (vitamin B12 binding protein, R binder family) | 2.056 | 0.020 |
|  |  |  |  | *HDC* | histidine decarboxylase | 2.043 | 0.015 |
|  |  |  |  | *NFIL3* | nuclear factor, interleukin 3 regulated | 2.017 | 0.036 |
|  | | | | | | | |
| *IMT (abnormal/high vs normal/low)* | | | | | | | |
|  | | | | | | | |
| *G0S2* | G0/G1 switch 2 | 3.036 | 0.027 | *PPP1CB* | protein phosphatase 1, catalytic β | 3.330 | 0.027 |
| *NRGN* | neurogranin | 3.007 | 0.008 | *HLADRB4* | MHC class II, DRβ 4 | 3.078 | 0.027 |
| *ITGA2B* | integrin α IIb / gpIIb/IIIa / CD41 | 2.702 | 0.008 | *TCEA1* | transcription elongation factor A 1 | 3.072 | 0.020 |
| *C3* | complement 3 | 2.554 | 0.015 | *IFNGR1* | interferon γ receptor 1 | 3.060 | 0.047 |
| *KLF3* | Kruppel-like factor 3 | 2.509 | 0.036 | *TMED2* | transmembrane emp24 domain trafficking protein 2 | 2.940 | 0.027 |
| *CLU* | clusterin | 2.502 | 0.011 | *CENPK* | centromere protein K | 2.914 | 0.047 |
| *FLNA* | filamin A, alpha | 2.477 | 0.006 | *RCBTB2* | BTB domain containing protein 2 | 2.894 | 0.020 |
| *SPATA20* | spermatogenesis associated 20 | 2.471 | 0.015 | *LRRN3* | leucine rich repeat neuronal 3 | 2.882 | 0.047 |
| *TCF7L2* | transcription factor 7-like 2 (T-cell specific, HMG-box) | 2.450 | 0.008 | *CCR2* | CC chemokine receptor 2 | 2.880 | 0.027 |
| *SLED1* | proteoglycan 3 pseudogene | 2.419 | 0.020 | *DCK* | deoxycytidine kinase | 2.816 | 0.027 |
| *C6orf25* | chromosome 6 open reading frame 25 | 2.418 | 0.006 | *CD46* | CD46 protein (complement regulation) | 2.812 | 0.036 |
| *PER1* | period homolog 1 (Drosophila) | 2.398 | 0.008 | *MBNL1* | musclebind-like splicing regulator 1 | 2.779 | 0.047 |
| *MGLL* |  | 2.394 | 0.004 | *ZNF420* | zinc finger protein 420 | 2.695 | 0.008 |
| *MYL9* | myosin, light chain 9, regulatory | 2.338 | 0.008 | *RTKN2* | rhotekin 2 | 2.663 | 0.011 |
| *PFN1* | profilin 1 | 2.329 | 0.027 | *IFI44L* | interferon-induced protein 44-like | 2.631 | 0.027 |
| *ARHGDIA* | Rho GDP dissociation inhibitor (GDI) alpha | 2.327 | 0.027 | *MS4A1* | membrane-spenning 4-domains A1 | 2.593 | 0.036 |
| *IRF5* | interferon regulatory factor 5 | 2.327 | 0.027 | *MORC3* | MORC family CW-type zinc finger 3 | 2.580 | 0.047 |
| *PLXNB2* | plexin B2 | 2.298 | 0.011 | *ELF1* | E74-like factor 1 (Ets domain transcription factor) | 2.549 | 0.036 |
| *CFL1* | cofilin 1 (non-muscle) | 2.298 | 0.020 | *IFIT1* | interferon-induced protein with tetratricopeptide repeats 1 | 2.546 | 0.047 |
| *POU2F2* | POU class 2 homeobox 2 | 2.285 | 0.006 | *VNN1* | vanin 1 | 2.526 | 0.027 |
| *GNAI2* | guanine nucleotide binding protein (G protein), alpha inhibiting activity polypeptide 2 | 2.268 | 0.004 | *PNRC2* | proline-rich nuclear receptor coactivator 2 | 2,454 | 0.047 |
| *PKN1* | protein kinase N1 | 2.264 | 0.027 | *HNRNPU-AS1* | HNRNPU antisense RNA 1 (non-protein coding) | 2.442 | 0.047 |
| *PNPLA6* | patatin-like phospholipase domain containing 6 | 2.259 | 0.008 | *NMD3* | NMD3 homolog (S. cerevisiae) | 2.440 | 0.008 |
| *ABCC3* | ATP-binding cassette, sub-family C (CFTR/MRP), member 3 | 2.246 | 0.011 | *PPM1B* | protein phosphatase, Mg2+/Mn2+ dependent, 1B | 2.401 | 0.036 |
| *TAGLN* | transgelin | 2.246 | 0.036 | *TLR10* | toll-like receptor 10 | 2.396 | 0.020 |
| *TP53I11* | tumor protein p53 inducible protein 11 | 2.213 | 0.027 | *TMEM30A* | transmembrane protein 30A | 2.395 | 0.036 |
| *CAPNS1* | calpain, small subunit 1 | 2.203 | 0.020 | *ARFIP1* | ADP-ribosylation factor interacting protein 1 | 2.384 | 0.027 |
| *SH3BGRL3* | SH3 domain binding glutamic acid-rich protein like 3 | 2.193 | 0.011 | *CD164* | CD164 molecule, sialomucin | 2.383 | 0.047 |
| *EHBP1L1* | EH domain binding protein 1-like 1 | 2.188 | 0.006 | *SNX10* | sorting nexin 10 | 2.382 | 0.036 |
| *SERPINA1* | serpin peptidase inhibitor, clade A (alpha-1 antiproteinase, antitrypsin), member 1 | 2.171 | 0.036 | *FTSJD1* | FtsJ methyltransferase domain containing 1 | 2.377 | 0.020 |
| *SPTAN1* | spectrin, alpha, non-erythrocytic 1 | 2.166 | 0.020 | *PID1* | phosphotyrosine interaction domain containing 1 | 2.357 | 0.015 |
| *AKT2* | v-akt murine thymoma viral oncogene homolog 2 | 2.153 | 0.015 | *GPR34* | G protein-coupled receptor 34 | 2.353 | 0.036 |
| *C5orf4* | chromosome 5 open reading frame 4 | 2.133 | 0.004 | *GATM* | glycine amidinotransferase (L-arginine:glycine amidinotransferase) | 2.351 | 0.003 |
| *IL2RG* | interleukin 2 receptor, gamma | 2.132 | 0.011 | *DEK* | DEK oncogene | 2.351 | 0.027 |
| *FAM101B* | family with sequence similarity 101, member B | 2.123 | 0.008 | *CCDC126* | coiled-coil domain containing 126 | 2.344 | 0.015 |
| *SPI1* | spleen focus forming virus (SFFV) proviral integration oncogene spi1 | 2.119 | 0.047 | *FAM200A* | family with sequence similarity 200, member A | 2.340 | 0.047 |
| *CCL4L1 /// CCL4L2* | chemokine (C-C motif) ligand 4-like 1 /// chemokine (C-C motif) ligand 4-like 2 | 2.106 | 0.006 | *PJA2* | praja ring finger 2, E3 ubiquitin protein ligase | 2.334 | 0.036 |
| *PLK3* | polo-like kinase 3 | 2.103 | 0.020 | *ZNF681* | zinc finger protein 681 | 2.319 | 0.004 |
| *UBA1* | ubiquitin-like modifier activating enzyme 1 | 2.100 | 0.036 | *MAT2B* | methionine adenosyltransferase II, beta | 2.318 | 0.036 |
| *TMEM158* | transmembrane protein 158 (gene/pseudogene) | 2.092 | 0.027 | *FMR1* | fragile X mental retardation 1 | 2.315 | 0.047 |
| *HNRNPUL1* | heterogeneous nuclear ribonucleoprotein U-like 1 | 2.083 | 0.020 | *IFIT2* | interferon-induced protein with tetratricopeptide repeats 2 | 2.307 | 0.027 |
| *AES* | amino-terminal enhancer of split | 2.081 | 0.008 | *SMAD4* | SMAD family member 4 | 2.305 | 0.036 |
| *CLPTM1* | cleft lip and palate associated transmembrane protein 1 | 2.076 | 0.047 | *FNDC3A* | fibronectin type III domain containing 3A | 2.301 | 0.027 |
| *TUBB2A* | tubulin, beta 2A class IIa | 2.072 | 0.006 | *DNAAF2* | dynein, axonemal, assembly factor 2 | 2.300 | 0.008 |
| *RHOG* | ras homolog family member G | 2.067 | 0.020 | *NABP1* | nucleic acid binding protein 1 | 2.299 | 0.011 |
| *ACTN1* | actinin, alpha 1 | 2.065 | 0.006 | *TM9SF3* | transmembrane 9 superfamily member 3 | 2.288 | 0.020 |
| *HLA-B /// HLA-C* | major histocompatibility complex, class I, B /// major histocompatibility complex, class I, C | 2.056 | 0.020 | *GSTM3* | glutathione S-transferase mu 3 (brain) | 2.278 | 0.036 |
| *TNFAIP3* | tumor necrosis factor, alpha-induced protein 3 | 2.053 | 0.011 | *CCNG1* | cyclin G1 | 2.278 | 0.036 |
| *ICAM1* | intercellular adhesion molecule 1 | 2.048 | 0.036 | *ZMPSTE24* | zinc metallopeptidase STE24 homolog (S. cerevisiae) | 2.277 | 0.036 |
| *MYO18A /// TIAF1* | myosin XVIIIA /// TGFB1-induced anti-apoptotic factor 1 | 2.040 | 0.015 | *DENND4A* | DENN/MADD domain containing 4A | 2.271 | 0.011 |
| *TRIM41* | tripartite motif containing 41 | 2.040 | 0.011 | *UFM1* | ubiquitin-fold modifier 1 | 2.271 | 0.011 |
| *MAP4K2* | mitogen-activated protein kinase kinase kinase kinase 2 | 2.028 | 0.008 | *HSDL1* | hydroxysteroid dehydrogenase like 1 | 2.269 | 0.036 |
| *CSNK1G2* | casein kinase 1, gamma 2 | 2.026 | 0.004 | *TMX1* | thioredoxin-related transmembrane protein 1 | 2.267 | 0.036 |
| *MAZ* | MYC-associated zinc finger protein (purine-binding transcription factor) | 2.019 | 0.036 | *CCNC* | cyclin C | 2.257 | 0.027 |
| *EMP3* | epithelial membrane protein 3 | 2.018 | 0.047 | *SACM1L* | SAC1 suppressor of actin mutations 1-like (yeast) | 2.255 | 0.027 |
| *TTC38* | tetratricopeptide repeat domain 38 | 2.015 | 0.006 | *NEDD1* | neural precursor cell expressed, developmentally down-regulated 1 | 2.254 | 0.036 |
| *KLF13* | Kruppel-like factor 13 | 2.014 | 0.002 | *ZBTB11* | zinc finger and BTB domain containing 11 | 2.249 | 0.047 |
| *PPP1R12C* | protein phosphatase 1, regulatory subunit 12C | 2.012 | 0.008 | *SEC23A* | Sec23 homolog A (S. cerevisiae) | 2.231 | 0.036 |
| *ATP6V0C* | ATPase, H+ transporting, lysosomal 16kDa, V0 subunit c | 2.009 | 0.036 | *OSTM1* | osteopetrosis associated transmembrane protein 1 | 2.228 | 0.011 |
| *CDKN1C* | cyclin-dependent kinase inhibitor 1C (p57, Kip2) | 2.005 | 0.027 | *STX12* | syntaxin 12 | 2.223 | 0.036 |
| *RUNX3* | runt-related transcription factor 3 | 2.005 | 0.020 | *FBXO22* | F-box protein 22 | 2.192 | 0.015 |
| *MYO1G* | myosin IG | 2.003 | 0.008 | *SGPP1* | sphingosine-1-phosphate phosphatase 1 | 2.187 | 0.027 |
|  | | | | *NUP43* | nucleoporin 43kDa | 2.184 | 0.027 |
|  |  |  |  | *ZNF655* | zinc finger protein 655 | 2.183 | 0.047 |
|  |  |  |  | *SERP1* | stress-associated endoplasmic reticulum protein 1 | 2.177 | 0.027 |
|  |  |  |  | *TRA2A* | transformer 2 alpha homolog (Drosophila) | 2.164 | 0.006 |
|  |  |  |  | *IFI44* | interferon-induced protein 44 | 2.162 | 0.020 |
|  |  |  |  | *TRIM23* | tripartite motif containing 23 | 2.161 | 0.020 |
|  |  |  |  | *ZNF655* | zinc finger protein 655 | 2.160 | 0.015 |
|  |  |  |  | *CPOX* | coproporphyrinogen oxidase | 2.158 | 0.004 |
|  |  |  |  | *PAPOLA* | poly(A) polymerase alpha | 2.141 | 0.027 |
|  |  |  |  | *RB1* | retinoblastoma 1 | 2.139 | 0.015 |
|  |  |  |  | *C15orf29* | chromosome 15 open reading frame 29 | 2.125 | 0.027 |
|  |  |  |  | *FNDC3B* | fibronectin type III domain containing 3B | 2.122 | 0.020 |
|  |  |  |  | *SUZ12* | suppressor of zeste 12 homolog (Drosophila) | 2.116 | 0.027 |
|  |  |  |  | *MST4* | serine/threonine protein kinase MST4 | 2.115 | 0.047 |
|  |  |  |  | *RNF141* | ring finger protein 141 | 2.115 | 0.036 |
|  |  |  |  | *CMPK1* | cytidine monophosphate (UMP-CMP) kinase 1, cytosolic | 2.108 | 0.027 |
|  |  |  |  | *KLRC1 /// KLRC2* | killer cell lectin-like receptor subfamily C, member 1 /// killer cell lectin-like receptor subfamily C, member 2 | 2.103 | 0.036 |
|  |  |  |  | *SS18* | synovial sarcoma translocation, chromosome 18 | 2.100 | 0.036 |
|  |  |  |  | *ETNK1* | ethanolamine kinase 1 | 2.097 | 0.036 |
|  |  |  |  | *CHURC1* | churchill domain containing 1 | 2.091 | 0.015 |
|  |  |  |  | *PKIB* | protein kinase (cAMP-dependent, catalytic) inhibitor beta | 2.090 | 0.003 |
|  |  |  |  | *NOC3L* | nucleolar complex associated 3 homolog (S. cerevisiae) | 2.089 | 0.008 |
|  |  |  |  | *CLK4* | CDC-like kinase 4 | 2.088 | 0.027 |
|  |  |  |  | *ABCD3* | ATP-binding cassette, sub-family D (ALD), member 3 | 2.085 | 0.020 |
|  |  |  |  | *ERBB2IP* | erbb2 interacting protein | 2.083 | 0.036 |
|  |  |  |  | *SRSF1* | serine/arginine-rich splicing factor 1 | 2.079 | 0.047 |
|  |  |  |  | *BTAF1* | BTAF1 RNA polymerase II, B-TFIID transcription factor-associated, 170kDa (Mot1 homolog, S. cerevisiae) | 2.078 | 0.047 |
|  |  |  |  | *SUB1* | SUB1 homolog (S. cerevisiae) | 2.077 | 0.047 |
|  |  |  |  | *PLXNC1* | plexin C1 | 2.075 | 0.027 |
|  |  |  |  | *PEX3* | peroxisomal biogenesis factor 3 | 2.074 | 0.027 |
|  |  |  |  | *CBLL1* | Cbl proto-oncogene, E3 ubiquitin protein ligase-like 1 | 2.072 | 0.020 |
|  |  |  |  | *PSAT1* | phosphoserine aminotransferase 1 | 2.070 | 0.008 |
|  | | | | *APPL1* | adaptor protein, phosphotyrosine interaction, PH domain and leucine zipper containing 1 | 2.070 | 0.006 |
|  |  |  |  | *PLSCR1* | phospholipid scramblase 1 | 2.068 | 0.027 |
|  |  |  |  | *LOC645405 /// OXR1* | oxidation resistance 1 pseudogene /// oxidation resistance 1 | 2.063 | 0.047 |
|  |  |  |  | *MAT2A* | methionine adenosyltransferase II, alpha | 2.061 | 0.011 |
|  |  |  |  | *AP5M1* | adaptor-related protein complex 5, mu 1 subunit | 2.057 | 0.020 |
|  |  |  |  | *ARID4B* | AT rich interactive domain 4B (RBP1-like) | 2.051 | 0.036 |
|  |  |  |  | *ZNF189* | zinc finger protein 189 | 2.048 | 0.047 |
|  |  |  |  | *PPT1* | palmitoyl-protein thioesterase 1 | 2.046 | 0.047 |
|  |  |  |  | *LEPROTL1* | leptin receptor overlapping transcript-like 1 | 2.042 | 0.036 |
|  |  |  |  | *RNFT1* | ring finger protein, transmembrane 1 | 2.038 | 0.020 |
|  |  |  |  | *KIAA1033* | KIAA1033 | 2.038 | 0.036 |
|  |  |  |  | *PTPLB* | protein tyrosine phosphatase-like (proline instead of catalytic arginine), member b | 2.036 | 0.047 |
|  |  |  |  | *METTL21D* | methyltransferase like 21D | 2.033 | 0.036 |
|  |  |  |  | *GYG1* | glycogenin 1 | 2.030 | 0.047 |
|  |  |  |  | *TMEM2* | transmembrane protein 2 | 2.028 | 0.047 |
|  |  |  |  | *PAPD4* | PAP associated domain containing 4 | 2.028 | 0.047 |
|  |  |  |  | *SPTLC1* | serine palmitoyltransferase, long chain base subunit 1 | 2.027 | 0.036 |
|  |  |  |  | *ZNF189* | zinc finger protein 189 | 2.026 | 0.047 |
|  |  |  |  | *RSAD2* | radical S-adenosyl methionine domain containing 2 | 2.026 | 0.047 |
|  |  |  |  | *PPM1K* | protein phosphatase, Mg2+/Mn2+ dependent, 1K | 2.025 | 0.027 |
|  |  |  |  | *LOC729020 /// RPE* | rcRPE /// ribulose-5-phosphate-3-epimerase | 2.024 | 0.011 |
|  |  |  |  | *SH2D1A* | SH2 domain containing 1A | 2.024 | 0.036 |
|  |  |  |  | *SLC25A46* | solute carrier family 25, member 46 | 2.021 | 0.015 |
|  |  |  |  | *SP3* | Sp3 transcription factor | 2.020 | 0.047 |
|  |  |  |  | *FAR1* | fatty acyl CoA reductase 1 | 2.018 | 0.036 |
|  |  |  |  | *BCLAF1* | BCL2-associated transcription factor 1 | 2.018 | 0.047 |
|  |  |  |  | *GOLPH3* | golgi phosphoprotein 3 (coat-protein) | 2.016 | 0.047 |
|  |  |  |  | *MIER3* | mesoderm induction early response 1, family member 3 | 2.014 | 0.027 |
|  |  |  |  | *EIF5* | eukaryotic translation initiation factor 5 | 2.012 | 0.015 |
|  |  |  |  | *ZNF140* | zinc finger protein 140 | 2.010 | 0.011 |
|  |  |  |  | *CCT2* | chaperonin containing TCP1, subunit 2 (beta) | 2.008 | 0.036 |
|  |  |  |  | *GNPDA2* | glucosamine-6-phosphate deaminase 2 | 2.008 | 0.027 |
|  |  |  |  | *CASD1* | CAS1 domain containing 1 | 2.005 | 0.027 |
|  |  |  |  | *C5orf28* | chromosome 5 open reading frame 28 | 2.003 | 0.020 |
|  |  |  |  | *EED* | embryonic ectoderm development | 2.001 | 0.011 |
|  | | | | | | | |
| *PWV (abnormal/high vs normal/low)* | | | | | | | |
|  | | | | | | | |
| *HLA-B /// HLA-C* | major histocompatibility complex, class I, B /// major histocompatibility complex, class I, C | 2.236 | 0.007 | *LLRN3* | leucine-rich repeat neuronal 3 | 2.967 | 0.023 |

FC(abs): absolute fold change. See manuscript for other abbreviations.
