# Supplementary material for: Gene expression analysis of vascular pathophysiology related to anti-TNF treatment in rheumatoid arthritis
Source: Arthritis Res Ther. 2019 Apr 15;21:94. doi: 10.1186/s13075-019-1862-6 (PMC6466794; doi:10.1186/s13075-019-1862-6)
Supplement: Supplementary file 2 — Table S2. Association of gene expression profiles with clinical and vascular responses upon anti-TNF therapy in RA patients (n = 19) (study 2). (DOCX 22 kb) [file 13075_2019_1862_MOESM2_ESM.docx]

**Table S2.** Association of gene expression profiles with clinical and vascular responses upon anti-TNF therapy in RA patients (n=19) (Study 2)^*^

| **Up-regulated genes (≥2-fold)** | | | | **Down-regulated genes (≥2-fold)** | | | |
| --- | --- | --- | --- | --- | --- | --- | --- |
| ***Gene symbol*** | ***Gene title*** | ***FC(abs)*** | ***p*** | ***Gene symbol*** | ***Gene title*** | ***FC(abs)*** | ***p*** |
|  | | | | | | | |
| *Clinical response (R vs NR)* | | | | | | | |
|  | | | | | | | |
| *HLADRB4* | MHC class II, DRβ 4 | 3.951 | 0.009 |  | | | |
| *TMEM176B* | transmembrane protein 176B | 2.656 | 0.028 |  |  |  |  |
| *IFI44* | interferon-induced protein 44 | 2.268 | 0.028 |  |  |  |  |
| *PLSCR1* | phospholipid scramblase 1 | 2.219 | 0.011 |  |  |  |  |
| *TMEM176A* | transmembrane protein 176A | 2.032 | 0.023 |  |  |  |  |
|  | | | | | | | |
| *FMD-20% (R vs NR)* | | | | | | | |
|  | | | | | | | |
| *NEFL* | neurofilament, light polypeptide | 2.038 | 0.031 | *JUN* | jun proto-oncogene | 2.316 | 0.022 |
|  | | | | *GYPB* | glycophorin B (MNS blood group) | 2.173 | 0.028 |
|  | | | | | | | |
| *IMT-20% (R vs NR)* | | | | | | | |
|  | | | | | | | |
| *LOC642838* | Igκ chain, V-I region, Walker-like | 4.284 | 0.011 | CXCL5 | CXC chemokine ligand 5 | 3.201 | 0.006 |
| *IGKC* | Igκ constant | 4.108 | 0.006 | TRPT1 | tRNA phosphotransferase 1 | 2.690 | 0.001 |
| *IGLV1-44* | Igλ variable 44 | 4.003 | 0.011 | GUCY1A3 | guanylate cyclase 1, soluble, α3 | 2.564 | 0.002 |
| *LOC100653210* | Igκ chain V-III region, VG-like | 3.538 | 0.018 | AKAP12 | A kinase (PRKA) anchor protein 12 | 2.278 | 0.014 |
| *IGHA1* | Ig heavi constant α1 | 2.717 | 0.027 | ITGB3 | integrin, beta 3 (platelet glycoprotein IIIa, antigen CD61) | 2.223 | 0.002 |
| *TNFRSF17* | TNF receptor superfamily, member 17 | 2.529 | 0.041 | SESN3 | sestrin 3 | 2.191 | 0.041 |
| *TMEM176B* | transmembrane protein 176B | 2.369 | 0.028 | GCOM1 /// MYZAP | GRINL1A complex locus 1 /// myocardial zonula adherens protein | 2.174 | 0.003 |
| *CD74* | CD74 molecule, major histocompatibility complex, class II invariant chain | 2.357 | 0.006 | NEFL | neurofilament, light polypeptide | 2.126 | 0.003 |
| *FCRL5* | Fc receptor-like 5 | 2.342 | 0.009 | HBQ1 | hemoglobin, theta 1 | 2.120 | 0.041 |
| *APOBEC3B* | apolipoprotein B mRNA editing enzyme, catalytic polypeptide-like 3B | 2.311 | 0,018 | MMD | monocyte to macrophage differentiation-associated | 2.089 | 0.003 |
| *CD79A* | CD79a molecule, immunoglobulin-associated alpha | 2.259 | 0.034 | RHOBTB1 | Rho-related BTB domain containing 1 | 2.074 | 0.004 |
| *MZB1* | marginal zone B and B1 cell-specific protein | 2.233 | 0.034 | PRRC2C | proline-rich coiled-coil 2C | 2.018 | 0.009 |
| *IGH@ /// IGHA2* | immunoglobulin heavy locus /// immunoglobulin heavy constant alpha 2 (A2m marker) | 2.200 | 0.041 |  | | | |
| *HLA-DQA1 /// LOC100507718 /// LOC100509457* | major histocompatibility complex, class II, DQ alpha 1 /// HLA class II histocompatibility antigen, DQ alpha 1 chain-like /// HLA class II histocompatibility antigen, DQ alpha 1 chain-like | 2.169 | 0.009 |  |  |  |  |
| *HLA-DPB1* | major histocompatibility complex, class II, DP beta 1 | 2.153 | 0.041 |  | | | |
| *IFITM3* | interferon induced transmembrane protein 3 | 2.077 | 0.007 |  |  |  |  |
| *CST3* | cystatin C | 2.027 | 0.034 |  |  |  |  |
| *IGLC1* | Immunoglobulin lambda constant 1 (Mcg marker) | 2.015 | 0.028 |  |  |  |  |
|  | | | | | | | |
| *PWV-20% (R vs NR)* | | | | | | | |
|  | | | | | | | |
| *IFNG* | interferon-γ | 2.574 | 0.032 | *HLAC* | MHC class I, C | 8.704 | 0.017 |
| *JUN* | jun proto-oncogene | 2.262 | 0.017 | *GNB4* | guanine nucleotide binding protein (G protein), beta polypeptide 4 | 2.116 | 0.013 |
| *CCL4L1 /// CCL4L2* | chemokine (C-C motif) ligand 4-like 1 /// chemokine (C-C motif) ligand 4-like 2 | 2.094 | 0.032 | *NRG1* | neuregulin 1 | 2.066 | 0.011 |
|  | | | | *NEFL* | neurofilament, light polypeptide | 2.046 | 0.004 |
|  |  |  |  | *FKBP5* | FK506 binding protein 5 | 2.018 | 0.008 |
|  | | | | | | | |
| *GVR-20% (R vs NR)* | | | | | | | |
|  | | | | | | | |
| *LOC642838* | Igκ chain, V-I region, Walker-like | 3.482 | 0.020 | *NEFL* | neurofilament, light polypeptide | 2.236 | 0.001 |
| *LOC100653210* | Igκ chain, V-III region, VG-like | 3.371 | 0.013 | *CES1 /// CES1P1 /// LOC100653057 /// LOC100653086* | carboxylesterase 1 /// carboxylesterase 1 pseudogene 1 /// liver carboxylesterase 1-like /// uncharacterized LOC100653086 | 2.163 | 0.013 |
| *IGKC* | Igκ constant | 3.219 | 0.017 |  | | | |
| *IGLV1-44* | Igλ variable 44 | 3.172 | 0.039 |  |  |  |  |
| *SCN3A* | sodium channel, voltage-gated, type III, α subunit | 2.593 | 0.039 |  |  |  |  |
| *IGHM* | immunoglobulin heavy constant mu | 2.452 | 0.039 |  |  |  |  |
| *IGJ* | immunoglobulin J polypeptide, linker protein for immunoglobulin alpha and mu polypeptides | 2.223 | 0.048 |  |  |  |  |
| *CD79A* | CD79a molecule, immunoglobulin-associated alpha | 2.193 | 0.017 |  |  |  |  |
| *VPREB3* | pre-B lymphocyte 3 | 2.191 | 0.026 |  |  |  |  |
| *POU2AF1* | POU class 2 associating factor 1 | 2.162 | 0.032 |  |  |  |  |
| *FCRL5* | Fc receptor-like 5 | 2.026 | 0.021 |  |  |  |  |

^*^See text for definitions. R: responder, NR: non-responder. FC(abs): absolute fold change. See manuscript for other abbreviations.
